# Critical band masking reveals the effects of optical distortions on the channel mediating letter identification

**DOI:** 10.3389/fpsyg.2014.01060

**Published:** 2014-09-30

**Authors:** Laura K. Young, Hannah E. Smithson

**Affiliations:** ^1^Department of Experimental Psychology, University of OxfordOxford, UK; ^2^Centre for Advanced Instrumentation, Department of Physics, Durham UniversityDurham, UK

**Keywords:** optical distortions, critical band masking, ocular aberrations, letter identification, spatial frequency channels, visual Strehl ratio

## Abstract

There is evidence that letter identification is mediated by only a narrow band of spatial frequencies and that the center frequency of the neural channel thought to underlie this selectivity is related to the size of the letters. When letters are spatially filtered (at a fixed size) the channel tuning characteristics change according to the properties of the spatial filter (Majaj et al., 2002). Optical aberrations in the eye act to spatially filter the image formed on the retina—their effect is generally to attenuate high frequencies more than low frequencies but often in a non-monotonic way. We might expect the change in the spatial frequency spectrum caused by the aberration to predict the shift in channel tuning observed for aberrated letters. We show that this is not the case. We used critical-band masking to estimate channel-tuning in the presence of three types of aberration—defocus, coma and secondary astigmatism. We found that the maximum masking was shifted to lower frequencies in the presence of an aberration and that this result was not simply predicted by the spatial-frequency-dependent degradation in image quality, assessed via metrics that have previously been shown to correlate well with performance loss in the presence of an aberration. We show that if image quality effects are taken into account (using visual Strehl metrics), the neural channel required to model the data is shifted to lower frequencies compared to the control (no-aberration) condition. Additionally, we show that when spurious resolution (caused by π phase shifts in the optical transfer function) in the image is masked, the channel tuning properties for aberrated letters are affected, suggesting that there may be interference between visual channels. Even in the presence of simulated aberrations, whose properties change from trial-to-trial, observers exhibit flexibility in selecting the spatial frequencies that support letter identification.

## 1. Introduction

In 1994 Solomon and Pelli used critical band masking to show that, despite letters being broadband stimuli, their identification is mediated by a single narrow band of spatial frequencies. Since their ideal observer model (based on the requirement to discriminate differences between letters) exhibited low-pass filtering characteristics, rather than bandpass characteristics as derived from the performance of human observers, it was suggested that the low-frequency fall-off of the human-derived filter represents a visual constraint upon letter identification. This account is in accordance with human observers' inability to identify severely low-pass filtered letters, such as those with optical blur. Similar results have also been found by other authors (Ginsburg, 1980; Parish and Sperling, 1991; Alexander et al., 1994; Chung et al., 2002a,b; Majaj et al., 2002; Oruç and Landy, 2009).

Majaj et al. (2002) further suggested that the center frequency of the band mediating letter identification was driven by the spatial frequencies available in the signal. In the presence of added, filtered visual noise observers persisted in using the same spatial frequency channel to identify letters rather than shifting channels to avoid the masking effects of the noise. Furthermore, when letters were filtered with a Gaussian bandpass (on a log-frequency scale) filter, the center frequency of the band mediating their identification scaled, although less than proportionally, with the center frequency of the filter. In addition to shifting the visual channel in response to filtering of the stimulus, Oruç and Landy (2009) also suggested that, when the masking noise that is added to the stimulus dominates the equivalent noise that is associated with the contrast sensitivity function of human observers (which describes how spatial frequencies are transmitted by the visual system), it is possible for an observer to switch visual channels, although not necessarily optimally.

Under natural viewing, images formed on the retina are affected by the optical quality of the eye and the distortion introduced is equivalent to filtering that image. In this paper we aim to quantify the effect that reduced image quality has on the mechanism of letter identification by considering the interaction between the spatial frequency filtering effects of optical aberrations and the spatial frequency demands of a letter identification task.

The optical quality of the human eye can be characterized by its optical transfer function (OTF), which quantifies the phase and contrast with which different spatial frequency components are transmitted. For an optically perfect eye, image quality is limited only by diffraction and the OTF is a linearly decreasing real-valued function with a cut-off frequency determined by the pupil diameter and the wavelength of light. However, real eyes are composed of imperfect optical components that introduce aberrations and these distort the wavefront of the incident light and blur the image formed on the retina. The wavefront error can now be routinely measured *in vivo* using a Shack-Hartmann aberrometer to quantify both the low order aberrations, such as defocus and astigmatism, as well as the higher-order aberrations that cannot easily be compensated with current vision correction aids.

The filtering properties of an aberration (quantified by its OTF) are different from the bandpass filters that Majaj et al. (2002) applied to letters in two respects. Firstly, the OTFs of real optical aberrations are not typically bandpass in nature but are non-monotonic and tend to attenuate high frequencies more than low frequencies. The second is that the OTFs of real optical aberrations (as opposed to Gaussian blur, for example) can be complex-valued functions, indicating spatial-frequency-dependent phase changes (quantified by the phase transfer function, PTF) in addition to spatial-frequency-dependent contrast changes (quantified by the modulation transfer function, MTF). These phase changes are an important consideration as they can have a significant impact on the spatial forms in an image. A π phase change, one that changes the polarity of the contrast at a particular spatial frequency, can be particularly disruptive to object recognition (Ravikumar et al., 2010) as it creates spurious resolution, which introduces additional contours. If, as suggested by Majaj et al. (2002), the visual filter mediating an identification task is selected from the signal, introduction of additional contours could bias channel selection to a sub-optimal band of frequencies.

Considering these points the following question arises—is the spatial frequency band mediating the task altered in the presence of an aberration? In this paper we use critical band masking to estimate the center frequency of the channel mediating letter identification and we measure how this is altered by three different types of aberration—defocus, coma and secondary astigmatism. We additionally mask some of the spurious resolution present in the images to look for changes in channel selection. Masking is achieved by adding (ideal) bandpass filtered noise to the stimuli and the contrast threshold elevation (from the no noise condition) for letter identification is measured for different noise center frequencies, giving a response profile. For each condition, the center frequency of the visual filter mediating the task is derived from this response profile.

There has been increasing interest in the relationship between the higher-order aberrations in the eye and visual performance. It has been shown that higher-order aberrations are detrimental to visual performance and that the reduction in performance varies between the types of aberration and their amplitudes (Applegate et al., 2002, 2003; Chen et al., 2005; Rocha et al., 2007; Zhao et al., 2009; Cheng et al., 2010; Rouger et al., 2010; Young et al., 2013b). Furthermore, higher-order aberrations can additionally affect higher-level visual tasks such as reading (Young et al., 2011), facial recognition (Ravikumar et al., 2010; Sawides et al., 2010) and viewing natural images (Sawides et al., 2010). It is clear that the effects of these aberrations vary between visual tasks (Pepose and Applegate, 2005) and therefore it is likely that the effects of spatial-frequency-dependent changes in the stimulus depend on the spatial frequency requirements of the task. Indeed we have shown that, at least in the case of letter-based tasks, visual performance is better predicted by a model that incorporates the spatial frequencies used by the visual system for a particular task, in addition to considering image-based changes (Young et al., 2013a). Aberrations that have a strong effect on the spatial frequencies that mediate letter identification are likely to degrade performance more than aberrations that have a strong effect on task-irrelevant frequencies. Our approach to date has been to assume that the visual channel mediating letter identification is invariant under changes in aberration type and magnitude. One of the motivations of the current study is to consider the interaction between image properties and the neural channel mediating letter identification. Specification of the eye's optics permits calculation of the retinal images formed from letter-stimuli and previous work provides estimates of the neural channels mediating letter identification but, here, we consider the interaction between these two determinants of letter-identification performance.

In this paper we report the results of two analyses that aim to quantify the optical effects and predict the channel-based effects. The first analysis employs a template-matching model, based on maximum values of cross-correlations between letters, which quantifies the similarity (“confusability”) of letters for a particular aberration. We use this analysis to show the spatial frequency demands of the task based purely on the stimulus and the result is consistent with the findings of Solomon and Pelli (1994), even in the presence of an aberration. The second analysis is based on a visual Strehl metric for predicting visual performance from a measure of the optical quality of the eye. Visual Strehl metrics usually calculate the ratio of the sum under the OTF weighted by the human neural contrast sensitivity function (NCSF) to that same weighted sum for a diffraction limited system (Thibos et al., 2004). In our modified version of the visual Strehl ratio (Young et al., 2013a) we weight the OTF by the spatial frequency band mediating the task, which for sharp letters we assumed to be a Gaussian profile (in log-frequency space) with a center frequency of 3 cycles per letter (which subtended 1° in our experiment) and a bandwidth of 1 octave (consistent with the findings of Solomon and Pelli, 1994). Here we report visual Strehl ratios calculated using the magnitude of the OTF, termed the VSMTF, and visual Strehl ratios explicitly incorporating phase via a multiplicative combination of the MTF and PTF, which we have termed the VS_*combined*_ (Young et al., 2013b). We additionally calculated the VSOTF, which uses the real part of the OTF, but this gave very similar results to the VSMTF.

We have previously used both template matching and visual Strehl analyses to successfully predict the increase in contrast threshold (from the no aberration condition) for letter recognition in the presence of the three types of aberration under investigation. For letters presented with an aberration, and in the absence of a noise mask, the increases in contrast threshold (from the no aberration condition) quantify the performance loss over the entire frequency spectrum of those letters. Since aberrations cause spatial-frequency-dependent modifications to the stimulus it is not unreasonable to expect that the increases in contrast threshold may be spatial-frequency dependent. In this study we use critical-band masking to limit the spatial frequencies available to the observer by masking a band of frequencies with noise. For letters presented with an aberration, and in the presence of noise, the increase in contrast threshold (from the no aberration condition) should be related to the contrast loss induced by the aberration only within the spatial frequency range that remains available to the observer. For frequencies at which the aberration has little effect we would expect the increase in contrast threshold to be small. It is therefore entirely possible that the shape of the response profiles that we measure, and consequently the center frequencies derived from them, can be accounted for by considering the spatial-frequency dependent filtering of the aberrations. In this paper, by introducing additional filtering steps to our model to represent the masking effects of the visual noise, we make a direct comparison between the predicted performance and our observers' performance. We suggest that comparisons between the response profiles derived from our model and those derived from human observers should separate signal-based effects (due to image quality degradation) from observer-dependent effects (due to changes in the visual channel). Effects that are not captured by the model imply additional adaptive visual behaviors on behalf of the observer, which themselves have implications for the development of more effective models.

Finally, we recalculate the visual Strehl ratio with an additional step to optimize the center frequency of the standard Gaussian weighting that we use to represent the visual filter mediating letter identification, in order to find the best fit to the observer-derived data. Assuming that the visual Strehl metric effectively predicts performance loss due to image quality degradation, optimizing the Gaussian weighting (representing the visual channel) should capture observer-dependent effects and indicate the channel center frequency that gives rise to the contrast threshold elevations that we measure.

## 2. Materials and methods

### 2.1. Letters

**Figure 1:**
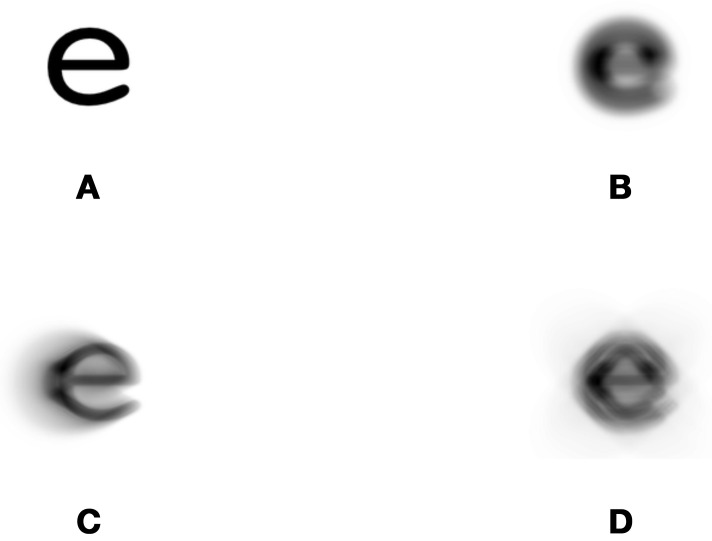
**Examples of the letter stimuli to which noise was added**. The control condition (i.e., no aberration) is shown in **(A)** and aberrated letters were generated with 0.6 μm of **(B)** defocus (Z^0^_2_), **(C)** coma (Z^1^_3_) or **(D)** secondary astigmastim (Z^2^_4_).

In a previous experiment we tested the effects of different amplitudes (0.5, 0.6, 0.7, 0.8, and 0.9 μm rms) of three types aberration (defocus, coma and secondary astigmatism) on observers' contrast thresholds for the identification of 1° letters (Young et al., 2013b). In the current experiment we have chosen to study the same three types of aberration but at a single amplitude—0.6 μm—at which observers showed a difference in performance between these three types of aberration. This amplitude corresponds to 2.7 D of equivalent defocus over a 2.5 mm diameter pupil. As in our previous experiment, single lower-case letter images were produced as black text on a light background (in this case a gray value of 0.5 was used whereas previously it had been 1.0) using Courier font. Images of aberrated letters, such as those shown in Figure 1, were generated using custom-written Python code that performed a convolution with the appropriate point spread function (PSF). We have made this code available to the community (see the Supplementary Materials).

As in our previous experiment, an unaberrated letter size of 1° of visual angle, corresponding to a Snellen acuity of 20/240, which is equivalent to 14 mm or 40 pt font at a typical reading distance of 40 cm, was chosen so that letter identification would be limited by the aberration and not by our observers' acuity limits. It is useful to consider how data collected at only one letter-size and one aberration amplitude might in principle generalize to other conditions. In the case of a bandpass filter applied to the letters, the center frequency of the filter is defined in cycles per letter so that when the size of a letter is changed the filtering effects remain consistent. However, whilst the OTF of an aberration can be specified as a function of frequency expressed in cycles per letter to give a consistent effect on a smaller letter, the result is not necessarily meaningful. The frequency scaling of real optical aberrations is determined by the wavelength of light and the diameter of the pupil, not by any simple combination of the size of the letters and the amplitude of the aberration. Changing the amplitude of the aberration does not simply re-scale the OTF along the frequency axis, it also changes its shape. In a previous experiment we compared the effects of an aberration at two different letter sizes (Young et al., 2013b). We used a normalized cross-correlation to find the amplitude of aberration that, when applied to a small letter, gave a similar stimulus appearance to a higher amplitude of aberration applied to a larger letter. Although the letters used in our experiments were large, and the amplitude of aberration was also correspondingly large, we know from our previous analysis that these stimuli are similar (correlation > 0.98) to 0.25° letters (corresponding to a Snellen acuity of 20/60, which is equivalent to 3.5 mm or 10 pt font at a typical reading distance of 40 cm) with an aberration amplitude of 0.25 μm rms. The aberration amplitudes found in the normal population are typically around 0.1 μm for horizontal coma and 0.05 μm for secondary astigmatism (Porter et al., 2001) though higher amplitudes are found in damaged or diseased eyes, such as those with keratoconus. Thus, the aberration amplitude we have used for large letters is high compared to the amplitudes found in the normal population, but the equivalent amplitude for small letters (at sizes typically encountered when reading text) is much closer to those normally found.

Majaj et al. (2002) showed that for filtered letters, changing the size of the letter alone gave a proportional relationship between the center frequency of the channel mediating letter identification and the size of the letter. We might expect that using a smaller letter size in our experiment would result in a proportional shift in the channel frequency that we measure for our observers. For unfiltered letters, as in the no aberration condition, the center frequency could be determined based on the stroke frequency of the letters, which they defined as the number of lines crossed by a horizontal slice through a letter, divided by the letter width, and averaged over all letters.

(1) fchannel10 cycles/degree=(fstroke10 cycles/degree)23.

The stroke frequency of the letters we used in the current experiment (1° Courier font letters) is 1.57 strokes per degree and we therefore expect the center frequency in the control condition to be 2.91 cycles per letter.

### 2.2. Noise

**Figure 2:**
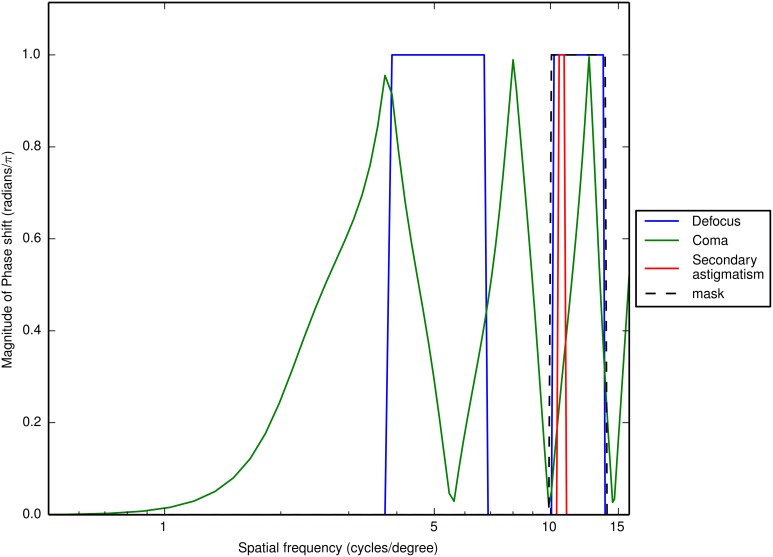
**The magnitude of the phase transfer functions for 0.6 μm of defocus (Z^0^_2_), coma (Z^1^_3_) and secondary astigmastim (Z^2^_4_)**. An additional band of noise was added centered at 11 cycles per degree with a bandwidth of 0.5 octaves (indicated by the dashed line) to coincide with spurious resolution created by defocus and secondary astigmatism. The phase shifts for coma are wrapped.

White noise samples were generated by using an array of pixel values (each 0.75 arcmin pixel contributed an individual noise check) that were sampled from a zero-mean Gaussian distribution with a standard deviation (rms contrast in this case) of 0.15 and truncated at two standard deviations. These white noise samples were bandpass filtered according to two classes of noise. The first part of the experiment was aimed at finding the center frequency of the channel mediating the identification of the aberrated letters. For this, noise samples were filtered with a one-octave-wide ideal bandpass filter centered at 1, 2, 3, 4, 6, 9, 11, or 14 cycles per degree. This was repeated with an additional bandpass filter to mask some of the spurious resolution. This additional filter was centered at 11 cycles per degree with a bandwidth of 0.5 octaves, determined by examining the OTFs of the three aberrations (see Figure 2). The final three conditions (9, 11 and 14 cycles per degree) were omitted when the additional mask was used as the noise-bands overlap. The entire noise field spanned 4° of visual angle and intensity values were scaled to a constant peak-to-valley contrast of 0.5 with a mean gray value of 0.5 (the same as the background of the letters).

### 2.3. Stimulus display

**Figure 3:**
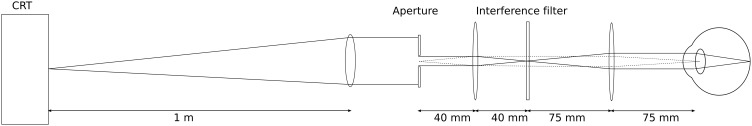
**The optical system used to relay the stimulus on the CRT to the observer's retina**. An aperture was used to control the observer's effective pupil size and an interference filter was used to reduce chromatic effects. The dotted line shows optical path relaying the aperture to the observer's pupil.

In these experiments blurred stimuli were created computationally via a convolution of a PSF with an image of a letter (see the Supplementary Materials). The resulting stimuli represent the image that would be formed on the retina by an eye with the specified PSF. To ensure that this was indeed the image formed on the retina of our observers it was necessary to consider the effects of aberrations introduced by the observer's eye and also to compensate for the effects of the display and the optics that relay the stimulus to the observer's eye (see Figure 3).

The resolution of an image formed on the retina is limited by the optical quality of the eye and the fundamental limit is that imposed by diffraction. Considering only the effects of diffraction it is best to use a large pupil to obtain the highest resolution images, however aberrations in the eye tend to increase with increasing pupil size. The optimal pupil size for lateral resolution has been shown to be 2.5 mm (Campbell and Green, 1965; Donnelly and Roorda, 2003), which is a diameter that has previously been used when simulating the effects of higher-order aberrations (see Cheng et al., 2010; Young et al., 2013b, for example). Additionally, we have measured the aberrations in our observers' eyes using a Zywave aberrometer and can confirm that over a 2.5 mm pupil they are close to diffraction-limited. Chromatic aberrations were avoided by using a narrowband interference filter centered at a wavelength of 550 nm.

The display and the optical system change the contrast of spatial frequencies in the image, quantified by their individual MTFs. The MTF of the entire optical system, including the display and the aperture, was measured via the slanted edge method (Estibeau and Magnan, 2004) using a camera that had been calibrated using the same technique in conjunction with an ISO 12233 test chart. This measure of the MTF of the optical system was used to pre-compensate the images for the contrast changes caused by the optical system.

The pre-compensated images were displayed on a CRT (Sony Trinitron, 1024 × 768 resolution) display using a Cambridge Research Systems VSG stimulus generator (VSG2/5) and the CRS Matlab toolboxes. To account for the intensity non-linearity in the display a gamma correction was applied to the stimuli using a look-up table, which was specified to maintain a resolution of 8 bits per gun for all stimulus contrasts, selected from 2^12^ available gray levels across the full intensity range. The letter image and the noise image were combined by temporally interleaving frames at a rate of 100 Hz. The mean luminance of the monitor measured through the optical system was 7.75 cd m^−2^.

Due to space constraints the image on the monitor had to be demagnified to maintain an acceptable sampling rate at the retina. In this arrangement, a single pixel on the display spanned 0.75 arc min on the retina, giving a sampling frequency of 80 pixels per degree. To prevent aliasing all stimuli were digitally low-pass filtered with a cut-off frequency of 40 cycles per degree. An aperture was used to artificially stop the pupil down to 2.5 mm and this was relayed to the eye's pupil using the optical system shown in Figure 3. This system produced a magnification factor of two between the artificial pupil and the observer's pupil, so an artificial pupil diameter of 1.25 mm was used. The cut-off frequency, *f_cut−off_*, of an optical system is defined by:

(2) $$f_{cut-off}=\frac{D}{\lambda },$$

where *D* is the diameter of the aperture and λ is the wavelength of light. The cut-off frequency in the intermediate focus, resulting from the aperture, was 40 cycles per degree. After demagnification this corresponded to a frequency of 80 cycles per degree at the retina, which was well above the cut-off frequency of the digital low-pass filter.

### 2.4. Procedure

The study received ethical approval from the Medical Sciences Division (MSD) Interdivisional Research Ethics Committee (IDREC) which operates under the Central University Research Ethics Committee (CUREC) at the University of Oxford. Informed consent was obtained from all observers. Three observers, two aged 28 and one aged 37, took part in the experiment. Two of the observers required refractive correction and so wore contact lenses. Observers were aligned to the instrument prior to beginning the experiment and were held in position by a chin rest. A separate rest was carefully positioned in front of the eye that observers could comfortably rest their cheek and brow bones against, allowing them to re-align themselves. Stimuli were displayed monocularly for 200 ms after which observers responded via a keyboard and audio feedback was then given.

For each trial, a letter was chosen at random and the probability of selection was weighted by the frequency counts of letters in the English language (Jones and Mewhort, 2004) in order to simulate natural reading conditions. The contrast of the noise was kept constant and Weber contrast thresholds for the letters were measured using the ML-PEST algorithm implemented with the Matlab Palamedes Toolbox (Prins and Kingdom, 2009). The algorithm converged to the threshold corresponding to 64% correct. Individual staircases for the experimental conditions were interleaved with each running for 20 trials per experimental condition and observers completed five sessions.

### 2.5. Prediction metrics

To investigate the effect of an aberration on image quality we used two types of metric, one based on template matching and one based on the visual Strehl ratio. In both cases we specifically considered the frequency-dependent changes, to consider the effect of the aberration on channel selection.

#### 2.5.1. Template matching

**Figure 4:**
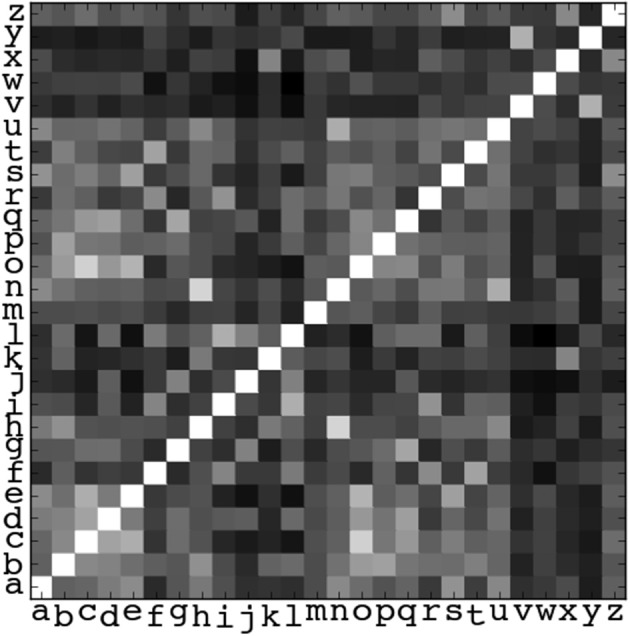
**An example confusion matrix indicating the maximum values of cross-correlations between letter images, in this case for the no aberration condition with no notch filter**. Gray values indicate values ranging from zero (black) to one (white). The matrix is normalized such that the values along the diagonal are equal to one. The confusability value associated with this matrix is 0.6.

Cross-correlation-based template matching models have been shown to have a high correlation with acuity measures (Watson and Ahumada, 2008, 2012, for example). We used a similar technique that we have previously shown to correlate well with empirical measures of performance in letter-based tasks (Young et al., 2011, 2013a,b). For the current experiment we added an additional filtering step to account for the masking effects of the noise. This technique made pair-wise comparisons between letters via a cross-correlation, as described in the following steps, which were repeated for each type of aberration and noise passband: (i) all of the letters of the alphabet were individually notch-filtered to remove the spatial frequencies that would be masked by noise in the experiment, (ii) the maximum of the cross-correlation between pairs of filtered letter images (one filtered, aberrated letter and one filtered, unaberrated letter) formed a 26-by-26 matrix, an example of which is given in Figure 4, (iii) the confusion matrix was normalized to one along the diagonal, (iv) the columns of the confusion matrix were weighted according the frequency with which letters appeared in the experiment, (v) the average value of the matrix was used as a measure of confusability between letters, (vi) confusability values were scaled such that a value of zero means that the only overlap is between a letter and its (unaberrated) template (i.e., the raw, unweighted correlation matrix is the identity matrix), and a confusability value of one means that all aberrated letters overlap with the unaberrated template by the same amount as the aberrated version of the template letter. The confusability value for unaberrated, unfiltered letters is 0.6. The confusion analysis was performed for comparisons between the aberrated letters and the unaberrated letter templates, as well as between pairs of aberrated letters. Similar results were obtained in both cases. Here we report only the comparisons between aberrated letters and the unaberrated letter templates.

#### 2.5.2. Visual strehl metrics

**Figure 5:**
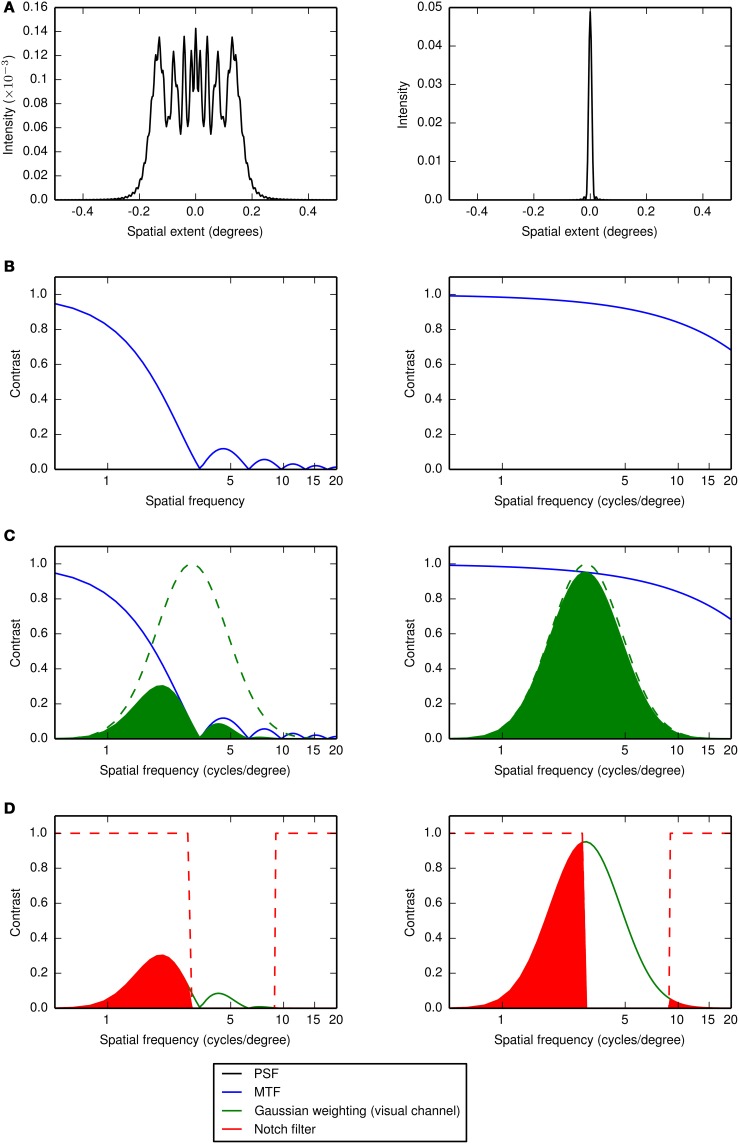
**The steps used to calculate the visual Strehl ratio (VSMTF in this example)**. The numerator in equation 3 is calculated as follows: For a particular aberration (shown on the left half of the figure) **(A)** the PSF is computed from the wavefront error associated with the aberration, **(B)** the MTF is computed as the Fourier transform of the PSF, **(C)** the MTF is weighted by a Gaussian function (in log frequency space and with mean equal to an observer's channel center frequency in the control condition) representing the visual channel mediating letter identification and **(D)** the result (green shaded area) is then additionally weighted by a notch filter that removes the spatial frequencies masked by the passband of noise. The result of this additional weighting (red shaded area) is then summed. The denominator in equation 3 is calculated by repeating steps **(A–D)** for a diffraction-limited PSF, as shown on the right half of the figure. The visual Strehl ratio is then the sum for the aberration divided by the sum for the diffraction-limited case and the result is weighted by the total signal power in the notch filter. A similar calculation is performed for the VS_*combined*_ (see Equation 4) except that in the numerator the MTF is weighted by the PTF, normalized between zero (at a phase shift of π) and one (at a phase shift of zero) and in the denominator the diffraction-limited MTF is replaced by the MTF for the aberration (Young et al., 2013a).

Using two types of visual Strehl metric (VSMTF and VS_*combined*_) we modeled the effect of the change of image quality on observers' performance. The visual Strehl ratio is a measure of (neurally weighted) relative image quality, quantifying the ratio of the sum under the OTF of an aberrated optical system to that of a diffraction limited one. In the traditional visual Strehl ratio (Thibos et al., 2004) the OTF is weighted by the human neural contrast sensitivity function, which attenuates high and very low spatial frequencies. We recently modified this metric such that the OTF is weighted by the neural filter that mediates the task (which for letter identification we had assumed to be a Gaussian function with a mean of 3 cycles per letter and a bandwidth of 1 octave) to give improved predictions of performance (Young et al., 2013a). In this paper we perform the same calculations but with two additional modifications. Firstly, for each observer, instead of using a Gaussian weighting, *LB*(*f_x_*, *f_y_*), with a mean of 3 cycles per letter we use a mean equal to the center frequency derived from the observer's performance in the control condition. Secondly, as for the template matching model, we introduce an additional notch filter, *NF*(*f_x_*, *f_y_*), to account for the masking effects of the noise. The equations representing the VSMF and VS_*combined*_ are

(3) $$VSMTF=\frac{\begin{array}{c} \int_{-\infty }^{\infty }\int_{-\infty }^{\infty }MTF\left(f_{x},f_{y}\right)\cdot LB\left(f_{x},f_{y}\right)\cdot \\ NF\left(f_{x},f_{y}\right)df_{x}df_{y} \end{array}}{\begin{array}{c} \int_{-\infty }^{\infty }\int_{-\infty }^{\infty }MTF_{DL}\left(f_{x},f_{y}\right)\cdot LB\left(f_{x},f_{y}\right)\cdot \\ NF\left(f_{x},f_{y}\right)df_{x}df_{y} \end{array}},$$

and

(4) $$VS_{combined}=\frac{\begin{array}{c} \int_{-\infty }^{\infty }\int_{-\infty }^{\infty }MTF\left(f_{x},f_{y}\right)\cdot \left(1-\frac{\left|PTF\left(f_{x},f_{y}\right)\right|}{\pi }\right)\cdot \\ LB\left(f_{x},f_{y}\right)\cdot NF\left(f_{x},f_{y}\right)df_{x}df_{y} \end{array}}{\begin{array}{c} \int_{-\infty }^{\infty }\int_{-\infty }^{\infty }MTF\left(f_{x},f_{y}\right)\cdot \\ LB\left(f_{x},f_{y}\right)\cdot NF\left(f_{x},f_{y}\right)df_{x}df_{y} \end{array}},$$

where *MTF*(*f_x_*, *f_y_*) is the MTF of the aberrated PSF, *MTF_DL_*(*f_x_*, *f_y_*) is the diffraction-limited MTF and *PTF*(*f_x_*,*f_y_*) is the PTF (in the range -π to π) of the aberrated PSF. A summary of the method for calculating the visual Strehl ratio is given in Figure 5 and further details can be found in our previous paper Young et al. (2013a).

As we have already shown that modified visual Strehl metrics are a good predictors of the increase in contrast threshold for letter identification from the no aberration condition, we would expect these results to closely match observers' change in noise-masked performance if observers continued to use the same band of spatial frequencies for each type of aberration. If this is the case we can assume that the change in the response profile (associated with an aberration) is caused by a reduction in image quality alone. If this is not the case, we wish to estimate which band of spatial frequencies our observers are using.

To estimate shifts in the putative neural channel underlying performance over and above changes imposed by the frequency-dependent changes in image quality, we additionally determine the center frequency of the Gaussian weighting (letter band, *LB*) with which to weight the OTF (as step Figure 5C), so as to minimize the sum of the squared differences between the visual Strehl ratio and the observer-derived increase in threshold (from the no aberration condition), within each particular noise band.

## 3. Results

### 3.1. Threshold elevation

For individual observers, threshold energy values, *E*, were calculated from the contrast threshold measure for each experimental condition and these were averaged over five sessions. These thresholds could be a result of intrinsic visual noise as well as the noise we have added. Therefore, we quote threshold elevations, *E*^+^ = *E* − *E*_0_, where *E*_0_ is the average threshold energy with no external noise. We also quote threshold signal-to-noise ratios, *SNR*, which are calculated as:

(5) $$SNR=\frac{E^{\;+}}{N},$$

where *N* is the noise power spectral density (Pelli and Farrel, 1999).

**Figure 6:**
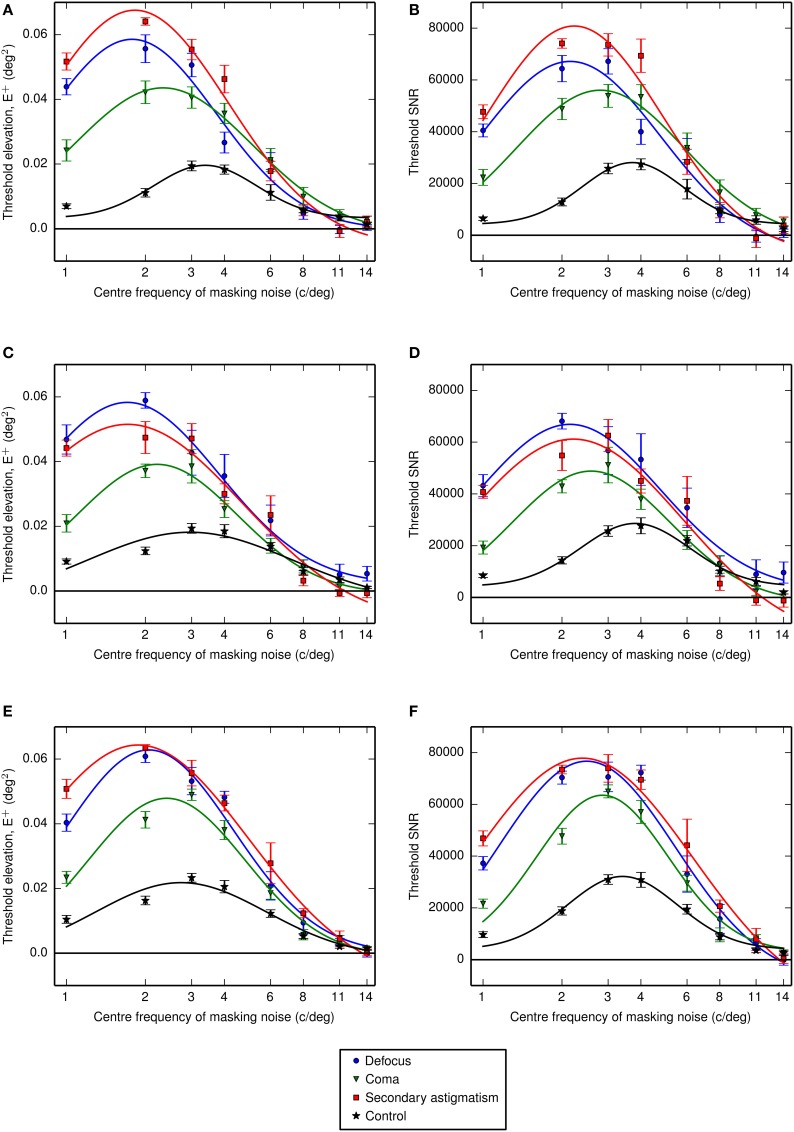
**Threshold elevations (above the no noise condition) for letter identification measured with noise that had been filtered using one-octave-wide (ideal) passbands at different center frequencies. (A,B)** Show the threshold energy elevations and threshold signal to noise ratios for observer LKY, **(C,D)** show the equivalent results for observer RJL and **(E,F)** for observer HES. The frequency axis is shown on a log_10_ scale and Gaussian functions were fitted to the data in log-frequency space. Error bars represent the standard error on the mean.

**Figure 7:**
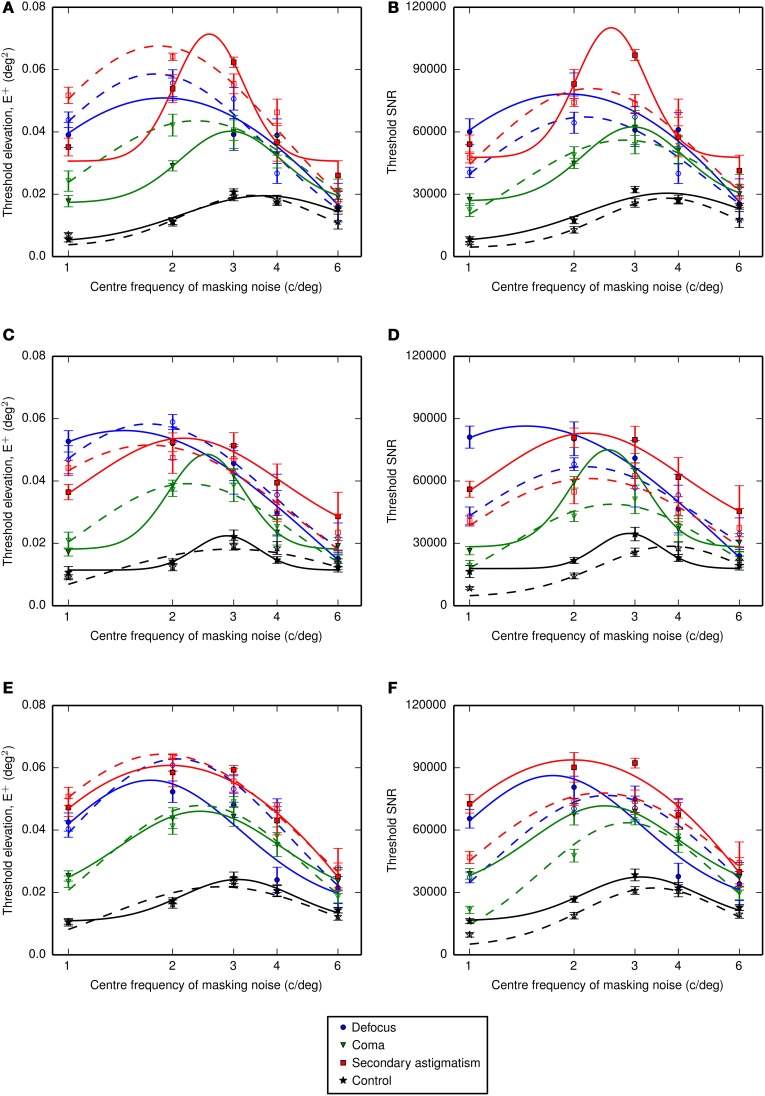
**Threshold elevations (above the no noise condition) for letter identification measured with noise that had been filtered using one-octave-wide (ideal) passbands at different center frequencies plus an additional band to mask spurious resolution in the letter images. (A,B)** Show the threshold energy elevations and threshold signal to noise ratios for observer LKY, **(C,D)** show the equivalent results for observer RJL and **(E,F)** for observer HES. The dashed lines with open symbols show the data in the absence of the spurious resolution mask, replotted from Figure 6. The frequency axis is shown on a log_10_ scale and Gaussian functions were fitted to the data in log-frequency space. Error bars represent the standard error on the mean.

Thresholds were obtained as a function of the center frequency of the one-octave ideal bandpass noise. The center frequency of the channel was estimated from the location of the maximum threshold elevation determined by fitting a Gaussian function (on a log-frequency scale) to the threshold signal-to-noise ratio and extracting the mean value. Thresholds were additionally obtained in the presence of an additional passband of noise designed to mask spurious resolution. The threshold elevations and signal-to-noise ratios are given in Figure 6 (simple bandpass mask) and Figure 7 (bandpass mask plus additional band to mask spurious resolution) and the center frequencies are summarized in **Figures 10A,B**.

Fitting Gaussian profiles to a measure of contrast threshold elevation gave an average center frequency (across observers) of 2.3 cycles per letter (standard error, SE = 0.1 cycles per letter) for defocus, 2.7 cycles per letter (SE = 0.1 cycles per letter) for coma, 2.3 cycles per letter (*SE* = 0.1 cycles per letter) for secondary astigmatism and 3.6 cycles per letter (*SE* = 0.1 cycles per letter) in the control condition. With the additional spurious resolution mask the average center frequencies were 1.7 cycles per letter (standard error, *SE* = 0.1 cycles per letter) for defocus, 2.6 cycles per letter (*SE* = 0.2 cycles per letter) for coma, 2.2 cycles per letter (*SE = 0.2* cycles per letter) for secondary astigmatism and 3.2 cycles per letter (*SE* = 0.2 cycles per letter) in the control condition.

These fitted center frequencies revealed a shift from the no aberration condition of between −0.45 and −0.82 octaves for defocus, between −0.25 and −0.56 octaves for coma and between −0.51 and −0.78 octaves for secondary astigmatism. The threshold signal-to-noise ratios show a consistent increase in threshold with the additional spurious resolution mask in the presence of an aberration (Figure 7). However, the spurious resolution mask only produced a significant shift (as determined by the 95% confidence limits) in center frequency for defocus for observers RJL and HES, and this was to lower frequencies. For observers LKY and HES, there is little difference between the control condition with the spurious resolution mask and the control condition without it, suggesting that this additional mask is having a negligible effect on performance. However, observer RJL exhibited a significant shift in the channel frequency for the control condition.

### 3.2. Predicting the channel

**Figure 8:**
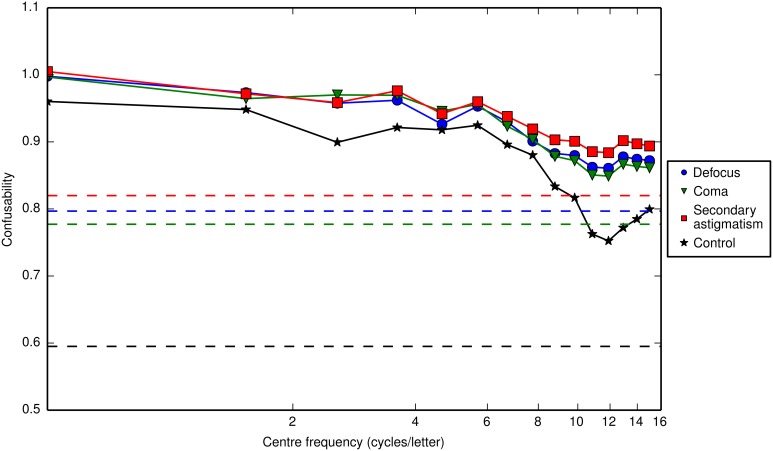
**The confusability of letters as a function of the center frequency of a one octave-wide notch filter applied to the letters**. A confusability value of zero means that the only overlap is between a letter and its (unaberrated) template (i.e., the raw, unweighted correlation matrix is the identity matrix), and a confusability value of one means that all the aberrated letters overlap to the same degree as they do with the (unaberrated) template (i.e., the raw, unweighted correlation matrix is a matrix of ones). A confusability value greater than one can be obtained, if on average, there is more overlap between an “incorrect” aberrated letter and the (unaberrated) template than there is between the “correct” aberrated letter and its (unaberrated) template. The confusability values for unfiltered letters are 0.60 in the control condition and 0.80, 0.78 and 0.82 for defocus, coma and secondary astigmatism respectively (indicated by the dashed lines). For all three types of aberration and the control, the confusability of letters is highest for low frequency notch filters. The notch filter removes a band of frequencies from the image, which we use to simulate bandpass filtered noise masking those same frequencies. The results suggest that the image characteristics that distinguish letters are concentrated at low spatial frequencies, since removing these frequencies increases confusability the most.

For unaberrated, unfiltered letters the confusability value is 0.60. For defocus, coma and secondary astigmatism the confusability values for unfiltered letter stimuli are 0.80, 0.78 and 0.82 respectively. Unsurprisingly, the aberrations increase confusability. But, of interest in our present experiment is how the distinguishing features that remain are distributed across the spatial frequency range. Figure 8 shows the relationship between confusability and the center frequency of the notch filter that had been applied to the letters to represent the masking effects of the bandpass noise. A high confusability value suggests that removing the band of frequencies (via filtering in our model or via masking in the experiment) makes letters more difficult to distinguish and therefore the identification task should be more difficult and the associated contrast thresholds correspondingly larger. The results show that, based purely on the demands of a template matching task, the response should be low pass, which is in agreement with the findings of Solomon and Pelli (1994). The low-pass characteristic found with unaberrated letters is retained for aberrated letters.

**Figure 9:**
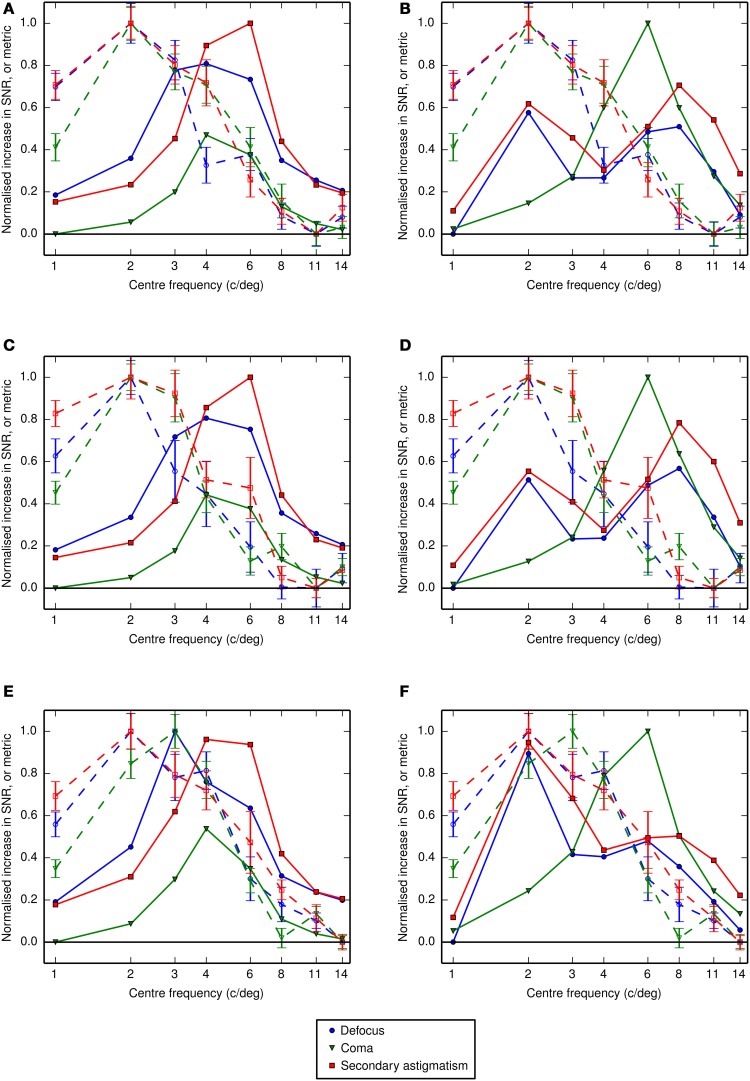
**(A,B)** Comparison between observer LKY's measured performance (dashed lines, open symbols) and a prediction of performance based on the visual Strehl ratio (solid lines, closed symbols) computed using **(A)** the VSMTF and **(B)** the VS_*combined*_. Panels **(C,D)** are the equivalent data for observer RJL and **(E,F)** are those for observer HES. As the visual Strehl ratio is high for good image quality the data are presented as the reciprocal of the visual Strehl ratio for comparison with threshold values. The observer-derived values reported here are the increase in threshold signal-to-noise ratio from the no aberration condition (i.e., the data presented as colored lines in the right half of Figure 6 minus the corresponding control data, presented as black lines), and in the absence of the additional spurious resolution mask. If our observers' performance were affected only by image quality degradation, and not a shift in the putative neural channel, the dashed lines should overlap with the solid lines.

To investigate the effects of a reduction in image quality caused by each aberration, we used the visual Strehl metrics described in Section 2.5.2. The visual Strehl ratios, shown in Figure 9, indicate the change in visual image quality associated with the addition of an aberration, within the band of frequencies we assume observers to be using for the task (based on their threshold elevations in the control condition). If a change in image quality were the only factor driving the difference in performance between aberrated and unaberrated letters, we would expect these values to correlate with the increases (from the no aberration condition) in threshold SNR measured empirically. Figure 9 shows that this is not the case.

**Figure 10:**
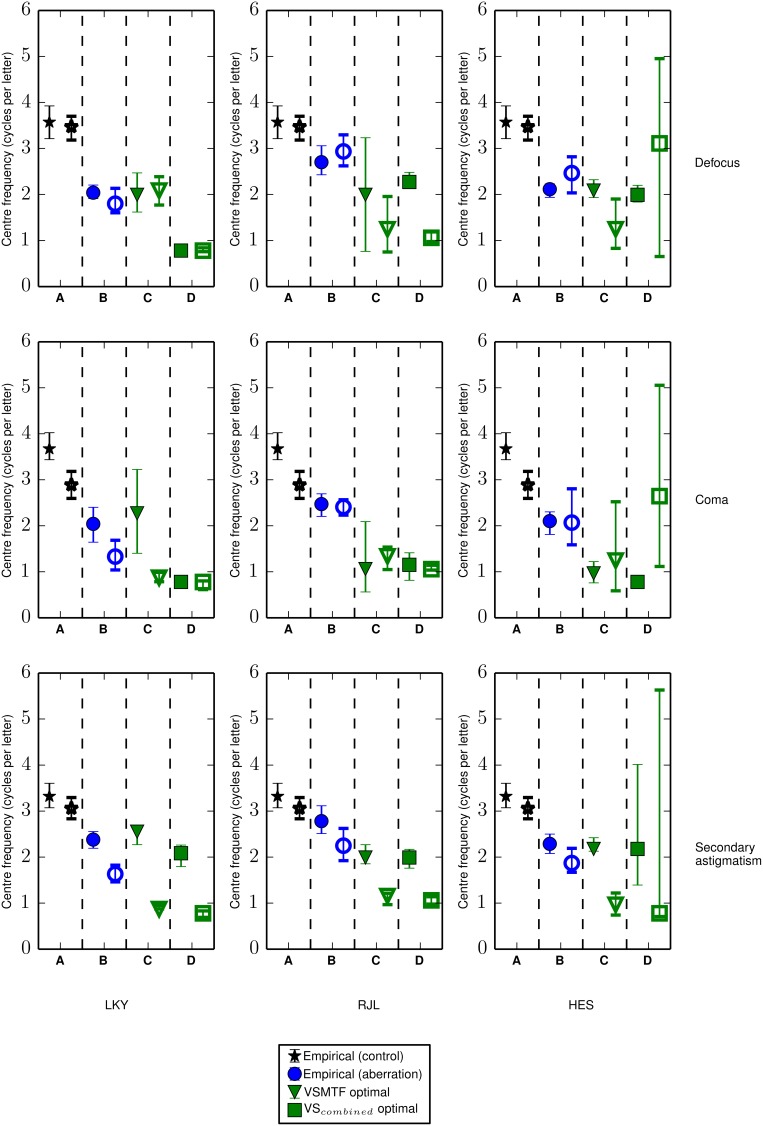
**The channel center frequencies derived from Gaussian fits (in log-frequency space) to the response profiles or by running the visual Strehl model**. Closed symbols show center frequencies in the absence of the spurious resolution mask and open symbols show the center frequencies with the additional spurious resolution mask. Data from different aberration types are shown in rows and data from different observers are shown in columns. Each sub-panel shows **(A)** the center frequency in the control condition (replotted in each row) derived from the empirical measure of threshold signal-to-noise ratio (as shown in right half of Figures 6, 7), **(B)** the similarly derived center frequency in the aberration condition, where the aberration type is indicated by the labels on the right of the figure, **(C)** the optimal center frequency for the Gaussian weighting (i.e., the putative visual channel) when calculating the VSMTF to most closely match the increase in the observer-derived threshold signal-to-noise ratio from the no aberration condition and **(D)** the optimal center frequency for the Gaussian weighting (i.e., the putative visual channel) when calculating the VS_*combined*_ to most closely match the increase in the observer-derived threshold signal-to-noise ratio from the no aberration condition. Error bars represent the 95% confidence limits, which were calculated by bootstrapping the data for each observer and each condition, drawing randomly with replacement from the data for individual trials, repeated for 1000 simulations.

To determine the weighting that should be applied to the OTF to represent the visual filter our observers are actually using in the aberration conditions, a sliding filter (as opposed to one generated from observers' measured center frequencies in the control condition) was used to reproduce the analysis summarized in Figure 5. Using this method we determined the center frequency of the sliding filter that produced visual Strehl ratios that most closely matched the observer-derived increase (from the no aberration condition) in threshold SNR. The results, given in Figures 10C,D, suggest that in the presence of an aberration the center frequency of the visual filter mediating letter identification is most likely lower than that for the control condition.

## 4. Discussion

When studying the effects of an optical aberration on visual performance it is important to consider not only the degradation of the image per se but specifically the loss of information in the image that is required to succeed at a visual task. Previous work has shown that visual performance is impaired in the presence of optical aberrations (Applegate et al., 2002, 2003; Chen et al., 2005; Rocha et al., 2007; Zhao et al., 2009; Cheng et al., 2010; Rouger et al., 2010; Young et al., 2013b). The reduction in visual performance is related to both the type of aberration and the amplitude of that aberration (Applegate et al., 2003, for example) and the effect is task-specific (Pepose and Applegate, 2005). Most work in this respect has focussed on letter-based tasks, most likely because they are a standard clinical optotype for assessing visual impairment and, being over-learned, they are ideal for testing object recognition. Aberrations cause spatial-frequency-dependent changes in an image and with letters being broadband stimuli we might expect that visual information is disrupted across the entire spectrum. However, any method for predicting performance that makes this assumption has the potential to incorrectly estimate the performance loss, since it will include degradation at spatial frequencies that do not ultimately mediate the task. Restricting the band of spatial frequencies over which a prediction metric is calculated to those mediating the task can mitigate this problem.

Solomon and Pelli (1994) showed that an ideal observer model predicts that the response profile, based purely on the demands of the task, should be low-pass. We additionally show that, even in the presence of aberrations, the response of an ideal observer (which we model via template matching) should also be low pass (Figure 8). Contrary to the predictions of their ideal observer model, Solomon and Pelli (1994) found, using critical band masking, that human observers use a single band of spatial frequencies centered at three cycles per letter with a bandwidth of one to two octaves. Further to this, Majaj et al. (2002) demonstrated that filtering a letter (at a fixed size) causes the center frequency to shift, scaling less than proportionally with the center frequency of the filter (defined in cycles per letter). Oruç and Landy (2009) also proposed that observers could switch spatial-frequency channels, but not necessarily to the optimal one. We hypothesized that real ocular aberrations, which act to spatially filter an image, could affect the spatial frequency channel mediating letter identification. Additionally, some aberrations create spurious resolution that introduces extra contours, which may drive the channel to a sub-optimal center frequency.

By using a critical-band masking technique for aberrated letter stimuli we have demonstrated measurable shifts in the channel frequency mediating their identification to lower frequencies. A shift from higher to lower spatial frequencies may be expected since aberrations generally attenuate higher frequencies but maintain contrast better at lower frequencies, yet confusability is less low pass in aberrated conditions than in the control (Figure 8). Across all observers there was a consistent trend showing that defocus and secondary astigmatism caused the largest shift in frequency band producing the peak masking effect and coma showed a smaller change.

Another possible effect could be that the bandwidth of the channel changes in the presence of an aberration if the information in the original channel is insufficient to identify the letter. We also tested the effects of changing the bandwidth of the noise by repeating the experiment with filtered noise having a varying bandwidth centered on the center frequencies determined for individual observers. These results are not presented here because they were too noisy to draw any firm conclusions from, most likely because the spatial frequency dependent contrast changes caused by these aberrations are non-monotonic. As the bandwidth increases it could potentially mask spurious resolution, or any other phase changes that may disrupt letter identification, and performance may partially improve. The results demonstrated a sigmoid shape but with additional dips that would be consistent with this hypothesis. However, as an approximation to the expected sigmoid shape it appeared that the bandwidth of the filter was not changing dramatically.

Ideally we would have compared thresholds measured in bandpass noise with those measured in notch filtered noise. This would have indicated whether observers were channel switching. While we did attempt this, any effects from the interaction between the notch filter and the aberration were lost in the noise due to having insufficient dynamic range for the noise contrast. We chose instead to look specifically at masking a secondary band of frequencies, coinciding with spurious resolution to investigate interactions between frequency channels.

If threshold performance depended only on a narrow band of frequencies, centered on 3 to 4 cycles per letter, adding the additional high-frequency mask should have no effect on performance. Our results on the other hand show a consistent increase in threshold with the additional mask (Figure 7) and in aberrated conditions there is a suggestion that the mask shifts the putative neural channel to lower frequencies (Figure 10B). Clearly there is some interaction between channels, although our results are insufficient to describe the nature of this interaction. The effects of spurious resolution are likely to be complex, perhaps producing false positives based on contours or features that might wrongly identify a letter, or driving looking to a suboptimal channel that has relatively high contrast. However, the way in which this is affected by the mask and by the observer's familiarity with the stimuli is beyond what we have tested here.

What is not clear from the data in Figures 6, 7 is whether the threshold elevations simply represent the residual information available in the noise-masked stimulus, or whether there is an interaction between the spatial frequencies used by the observer and the nature of the aberration. If observers persist in using the same visual channel in the presence of an aberration, we should be able to predict the change in threshold simply from the change in image quality within that channel. We have chosen to use visual Strehl metrics for this analysis. Our conclusions are dependent on these metrics providing an adequate summary of image quality, which is supported by several studies showing that they work well for predicting performance on letter-based tasks in the presence of an aberration (e.g., Marsack et al., 2004; Thibos et al., 2004; Young et al., 2013b). Using the center frequencies measured for the control condition we calculated the visual Strehl ratio in each noise filter band. Figure 9 shows how the increase in threshold SNR (from the no aberration condition) of our observers should be skewed if their performance were affected only by image quality degradation in the absence of any change in the visual channel they were using. It is clear that the values predicted from the visual Strehl ratio do not match the observer-derived increases in threshold SNR and we therefore assume that the our observers are using a different band of spatial frequencies than those derived in the control condition.

The response profile derived from an observer's threshold SNR in the control condition can be used to infer the center frequency of the neural channel associated with letter recognition for an optically ideal visual system. The corresponding response profiles measured in the aberration conditions include the effects of changes in image quality and any changes in the putative neural channel supporting performance. We use a modified visual Strehl ratio to capture the changes in the image (from the no-aberration condition) and the changes in the neural channel by finding the neural filter that, when used as the weighting function in the visual Strehl calculation, gives the best match between the metric and the measured increases in threshold SNR. We consider aberration-induced changes in the putative channel mediating letter identification to be the shifts in center frequency from the no-aberration condition (in which there are no image quality effects) to the center frequency of the sliding filter that most closely captures the measured performance changes. Figures 10C,D summarizes these results. The VSMTF reveals shifts in the putative visual channel to much lower spatial frequencies (as compared to the control condition, panel in Figure 10A), and this was also observed in the presence of the additional spurious resolution mask. The VS_*combined*_ metric produces inconsistent results across observers and this is most likely due to the non-monotonic nature of the VS_*combined*_ profiles. The non-monotonic profiles arise due to additional π phase changes at lower spatial frequencies (e.g., see Figure 2). Interestingly, these have a substantial effect on the metric but not on performance. Accurately modeling the consequences of phase changes for visual performance is difficult, and these results suggest the VS_*combined*_ metric should be further refined.

It is important to note that our observers were not adapted to these aberrations, as they might be if they were permanent feature of their vision. Therefore we cannot be sure that an observer with these aberrations occurring naturally would have a shifted center frequency with respect to the normal population. Additionally we have tested these three types of aberration in isolation whereas in a normal eye there would be combination of aberration types and amplitudes.

Our results suggest that the impact optical aberrations have on letter identification performance is not only based upon a loss of contrast or changes in phase but that there is also the potential for them to alter the neural channel selected to support letter identification. We already know that optical aberrations can have far reaching effects on visual performance as we have shown that certain types of aberration specifically affect the process of word recognition (Young et al., 2011) with uncommon words taking disproportionally longer to identify than common words in the presence of defocus or secondary astigmatism than with no aberration applied. Majaj et al. (2002) suggested that the channel mediating letter identification is selected bottom-up by the signal and our results broadly agree with this hypothesis in that observers' response profiles changed when an aberration was present. The measured change in performance was not simply predicted by the spatial-frequency-dependent change in image quality within a fixed channel, at least based on the image quality metrics we have used, suggesting that observers exhibit flexibility in the channel they select for letter identification in the presence of an aberration.

## Funding

We would like to acknowledge the John Fell Fund for supporting this work.

### Conflict of interest statement

The authors declare that the research was conducted in the absence of any commercial or financial relationships that could be construed as a potential conflict of interest.

## References

[B1] Alexander K. R. Xie W. Derlacki D. J. (1994). Spatial-frequency characteristics of letter identification. J. Opt. Soc. Am. A 11, 2375–2382 10.1364/JOSAA.11.002375 7931762

[B2] Applegate R. A. Ballentine C. Gross H. Sarver E. J. Sarver C. A. (2003). Visual acuity as a function of Zernike mode and level of root mean square error. Optom. Vis. Sci. 80, 97–105 10.1097/00006324-200302000-00005 12597324

[B3] Applegate R. A. Sarver E. J. Khemsara V. (2002). Are all aberrations equal? J. Refract. Surg. 18, S556–S562 12361157 10.3928/1081-597X-20020901-12

[B4] Campbell F. W. Green D. G. (1965). Optical and retinal factors affecting visual resolution. J. Physiol. 181, 576–593 5880378 10.1113/jphysiol.1965.sp007784 PMC1357668

[B5] Chen L. Singer B. Guirao A. Porter J. Williams D. R. (2005). Image metrics for predicting subjective image quality. Optom. Vis. Sci. 82, 358–369 10.1097/01.OPX.0000162647.80768.7F 15894912

[B6] Cheng X. Bradley A. Ravikumar S. Thibos L. N. (2010). Visual impact of Zernike and Seidel forms of monochromatic aberrations. Optom. Vis. Sci. 87, 300–312 10.1097/OPX.0b013e3181d95217 20351600 PMC3144141

[B7] Chung S. T. L. Legge G. E. Tjan B. S. (2002a). Spatial-frequency characteristics of letter identification in central and peripheral vision. Vis. Res. 42, 2137–2152 10.1016/S0042-6989(02)00092-5 12207975

[B7b] Chung S. T. L. Levi D. M. Legge G. E. (2002b). Spatial-frequency properties of letter identification in amblyopia. Vis. Res. 42, 1571–1581 10.1016/S0042-6989(02)00065-2 12074951

[B9] Donnelly W. J. Roorda A. (2003). Optimal pupil size in the human eye for axial resolution. J. Opt. Soc. Am. A 20, 2010–2015 10.1364/JOSAA.20.002010 14620328

[B10] Estibeau M. Magnan P. (2004). Fast mtf measurement of cmos imagers using iso 12233 slanted-edge methodology. Proc. SPIE 5251, 243–251 10.1117/12.513320

[B11] Ginsburg A. P. (1980). Specifying relevant spatial information for image evaluation and display design: an explanation of how we see certain objects. Proc. SID 21, 219–227

[B12] Jones M. N. Mewhort D. J. K. (2004). Case-sensitive letter and bigram frequency counts from large-scale English corpora. Behav. Res. Methods Instrum. Comput. 36, 388–396 10.3758/BF03195586 15641428

[B13] Majaj N. J. Pelli D. G. Kurshan P. Palomares M. (2002). The role of spatial frequency channels in letter identification. Vis. Res. 42, 1165–1184 10.1016/S0042-6989(02)00045-7 11997055

[B14] Marsack J. D. Thibos L. N. Applegate R. A. (2004). Metrics of optical quality derived from wave aberrations predict visual performance. J. Vis. 4, 322–328 10.1167/4.4.8 15134479

[B15] Oruç I. Landy M. S. (2009). Scale dependence and channel switching in letter identification. J. Vis. 9, 1–19 10.1167/9.9.4 19761337 PMC2775064

[B16] Parish D. H. Sperling G. (1991). Object spatial frequencies, retinal spatial frequencies, noise and the efficiency of letter discrimination. Vis. Res. 31, 1399–1415 10.1016/0042-6989(91)90060-I 1891827

[B17] Pelli D. G. Farrel B. (1999). Why use noise? J. Opt. Soc. Am. A 16, 647–653 10.1364/JOSAA.16.000647 10069051

[B18] Pepose J. S. Applegate R. A. (2005). Making sense out of wavefront sensing. Am. J. Ophthalmol. 139, 335–343 10.1016/j.ajo.2004.11.010 15733998

[B19] Porter J. Guirao A. Cox I. G. Williams D. R. (2001). Monochromatic aberrations of the human eye in a large population. J. Opt. Soc. Am. A 18, 1793–1803 10.1364/JOSAA.18.001793 11488483

[B20] Prins N. Kingdom F. A. A. (2009). Palamedes: Matlab Routines for Analyzing Psychophysical Data. Available online at: http://www.palamedestoolbox.org

[B21] Ravikumar S. Bradley A. Thibos L. N. (2010). Phase changes induced by optical aberrations degrade letter and face acuity. J. Vis. 10:18 10.1167/10.14.18 21163955

[B22] Rocha K. M. Vabre L. Harms F. Chateau N. Krueger R. R. (2007). Effect of Zernike wavefront aberrations on visual acuity measured using electromagnetic adaptive optics technology. J. Refract. Surg. 23, 953–959 18041253 10.3928/1081-597X-20071101-17

[B23] Rouger H. Benard Y. Legras R. (2010). Effect of monochromatic induced aberrations on visual performance measured by adaptive optics technology. J. Refract. Surg. 26, 578–587 10.3928/1081597X-20090901-01 19731885

[B24] Sawides L. Gambra E. Pascual D. Marcos S. (2010). Visual performance with real-life tasks under Adaptive-Optics ocular aberration correction. J. Vis. 10, 1–12 10.1167/10.5.19 20616133

[B25] Solomon J. A. Pelli D. G. (1994). The visual filter mediating letter identification. Nature 369, 395–397 10.1038/369395a0 8196766

[B26] Thibos L. N. Hong X. Bradley A. Applegate R. A. (2004). Accuracy and precision of objective refraction from wavefront aberrations. J. Vis. 4, 329–351 10.1167/4.4.9 15134480

[B27] Watson A. B. Ahumada A. J. (2008). Predicting visual acuity from wavefront aberrations. J. Vis. 8:17 10.1167/8.4.17 18484856

[B28] Watson A. B. Ahumada A. J. (2012). Modelling acuity for optotypes varying in complexity. J. Vis. 12:19 10.1167/12.10.19 23024356

[B29] Young L. K. Liversedge S. P. Myers R. M. Love G. D. Smithson H. E. (2011). Not all aberrations are equal: Reading impairment depends on aberration type and magnitude. J. Vis. 11:20 10.1167/11.13.20 22108058

[B30] Young L. K. Love G. D. Smithson H. E. (2013a). Accounting for the phase, spatial frequency and orientation demands of the task improves metrics based on the visual strehl ratio. Vis. Res. 90, 57–67 10.1016/j.visres.2013.06.007 23876993

[B31] Young L. K. Love G. D. Smithson H. E. (2013b). Different aberrations raise contrast thresholds for single-letter identification in line with their effect on cross-correlation-based confusability. J. Vis. 13:12 10.1167/13.7.12 23788460

[B32] Zhao H.-X. Xu B. Li J. Dai Y. Zhang Y.-D. Jiang W.-H. (2009). Effects of different Zernike terms on optical quality and vision of human eyes. Chin. Phys. Lett. 26:054205 10.1088/0256-307X/26/5/05420518427592

