# Supplementary material for: Critical band masking reveals the effects of optical distortions on the channel mediating letter identification
Source: Front Psychol. 2014 Sep 30;5:1060. doi: 10.3389/fpsyg.2014.01060 (PMC4179702; doi:10.3389/fpsyg.2014.01060)
Supplement: Supplementary file 1 [file Presentation1.PDF]

Frontiers in Perception

Research Article
28 July 2014

Supplementary Material: Critical band masking reveals the effects of optical distortions on the channel mediating letter identification

Laura K. Young [1,2,*] **and Hannah E. Smithson** [1]

[1] *Department of Experimental Psychology, University of Oxford, Oxford, UK*

Correspondence*:
Laura K. Young
[2]Centre for Advanced Instrumentation, Department of Physics, University of Durham, Durham, UK, laura.young@durham.ac.uk

Using noise to characterize vision

We have made the custom Python code for rendering aberrated stimuli available to the community (*http://sourceforge.net/projects/aberrationrendering/*).The code generates a point spread function (PSF) from an aberration (wavefront error) map and then convolves it with an image. In the following sections we break the task of rendering an aberrated stimulus into three parts- wavefront generation, PSF generation and convolution. For each part we provide background information with the relevant equations and notes on the implementation of those equations. Interactive examples are given in an iPython notebook file (*http://sourceforge.net/projects/aberrationrendering/*).

1 BACKGROUND

1.1 GENERAL BACKGROUND

Light emitted from a point source has a spherical wavefront and this wavefront defines a surface over which the propagating light wave has a constant phase. If that point source is located infinitely far away, the wavefront will be flat. As the light from an object at an infinite distance is focussed by an optical system, such as the eye, the planar wavefront is transformed into a spherical wavefront. The centre of curvature of that spherical wavefront is coincident with the corresponding point in the image plane where the light is brought to a focus. The mapping from a single point on the object to the corresponding point in the image plane is quantified by the PSF (i.e. the impulse response function) of the optical system. If the PSF is space invariant, the output intensity pattern is a linear superposition of the outputs from the individual points on the object. The field of view over which the PSF is spatially invariant is known as the isoplanatic patch and in the eye (at the fovea) this is approximately 1 to 2° (see **Bedgood et al.**, 2008, for a summary). For incoherent illumination, the output intensity pattern (i.e. the image) is a convolution of the input intensity pattern (i.e. from the object) with the PSF.

If an optical system is perfect, the PSF is limited only by diffraction at the aperture (in the eye this is the pupil). Imperfections in the optical system cause the wavefront to deviate from its ideal shape (e.g. spherical for a focussing system) and this wavefront error results in a degradation in image quality.

1.2 WAVEFRONT REPRESENTATION

Aberrations measured in the eye are typically decomposed into Zernike polynomials, which are an orthogonal basis set defined over a unit circle, and are quantified by the corresponding Zernike coefficients. Any wavefront aberration, *W(r, θ)*, can be represented with a Zernike polynomial expansion,

$$W(r,\theta) = \sum_{n_{\min}}^{n_{\max}} \sum_{m=-n}^{n} c_n^m Z_n^m(r,\theta), \tag{1}$$

where r is the radial coordinate, θ is the angular coordinate, n is the radial order, m is the angular order and c_n^m is the Zernike coefficient corresponding to the Zernike mode $Z_n^m(r,\theta)$. The number of radial orders used to represent the wavefront is determined by the minimum, $n_{\min}$, and maximum, $n_{\max}$, radial order values. The polynomials describing these Zernike modes are defined by

$$Z_n^m(r,\theta) = \begin{Bmatrix} N_n^m R_n^{|m|}(r)\cos m\theta & \mathit{for}\ m \geq 0 \\ N_n^m R_n^{|m|}(r)\sin m\theta & \mathit{for}\ m < 0 \end{Bmatrix}, \tag{2}$$

where $R_n^{|m|}(r)$ is the radial component given by

$$R_n^{|m|}(r) = \sum_{s=0}^{(n-|m|)/2} \frac{(-1)^s(n-s)!}{s![0.5(n+|m|-s)]![0.5(n-|m|-s]!} r^{n-2s}. \tag{3}$$

The normalisation constant is given by

$$N_n^m = \sqrt{\frac{2(n+1)}{1+\delta_{m0}}}, \tag{4}$$

where δ_{m0} is the Kronecker delta function ($\delta_{m0} = 1$ for $m = 0$ and $\delta_{m0} = 0$ for $m \neq 0$). We use the standard double index scheme, (itn,m), for describing Zernike terms but this can be converted to a single index, j, using the standard single indexing scheme defined by **Thibos et al.** (2000),

$$j = \frac{n(n+2)+m}{2}. \tag{5}$$

The Zernike coefficients give the amplitude of each Zernike mode in terms of optical path difference (expressed in units of length) and in ophthalmology, the Zernike coefficients are normally expressed in μm rms. Zernike coefficients are defined over a unit circle, but they can be converted to equivalent defocus if the pupil radius, r_{pupil}, is known. Equivalent defocus, M_e, is defined as the amount of defocus, in Diopters, that produces the same wavefront variance, RMS, as a given aberration,

$$M_e = \frac{4\sqrt{3}\,RMS}{r_{pupil}^2}. \tag{6}$$

1.3 PSF GENERATION

The PSF of an optical system is the Fourier transform, $\mathcal{F}$, of the electric field distribution across its aperture, which is known as the aperture function. For incoherent illumination we can compute the

incoherent PSF, $P(r,\theta)$, which has an intensity distribution equal to the squared modulus of the Fourier transform of the aperture function,

$$P(r,\theta) = |\mathcal{F}(p(r,\theta)e^{-i\frac{2\pi}{\lambda}W(r,\theta)})|^2, \tag{7}$$

where λ is the wavelength of light. The aperture function is complex because it has both a phase component and an amplitude component. The phase component is quantified by the wavefront error in the pupil, as discussed in Section 1.1, and the amplitude component is quantified by the transmission function of the pupil, $p(r,\theta)$. For the eye we can consider a circular, uniformly transmissive aperture, which has an amplitude component that is a circular mask with a value of one inside and a value of zero outside. Other amplitude functions can be used to simulate different aperture shapes but, more usefully, they can be used to simulate a non-uniformly transmissive pupil, such as might be found in an eye with a cataract, or to approximate the Stiles-Crawford effect.

The PSF is always normalised such that the total intensity is equal to one, since aberrations simply act to redistribute intensity, rather than diminish it.

1.4 CONVOLUTION OF THE PSF WITH AN INPUT INTENSITY PATTERN

As discussed in Section 1.1, the output intensity pattern, $I(r,\theta)$, of an optical system is the convolution of the input signal, $I(r,\theta)$, and the system's PSF,

$$\begin{aligned} I(r,\theta) \otimes P(r,\theta) &= \int\int I(\mu,\eta)P(r-\mu,\theta-\eta)d\mu d\eta \\ &= \mathcal{F}^{-1}[\mathcal{F}[I(r,\theta)]\mathcal{F}[P(r,\theta)]], \end{aligned} \tag{8}$$

where $\otimes$ indicates convolution and μ and η are the radial and angular frequency coordinates respectively.

It is important that the pixel scale in the PSF is matched to the pixel scale in the input intensity pattern. The pixel scale in the PSF is given by

$$s_{PSF} = \frac{1.22\lambda}{2r_{pupil}\alpha}, \tag{9}$$

where α is the oversampling factor (see Section 2.1.1). The pixel scale of the object is

$$s_{object} = \frac{\upsilon_{object}}{N_{object}}, \tag{10}$$

where υ_{object} is the field of view of the object and N_{object} is the number of pixels in that field of view. Combining Equations 9 and 10 and re-arranging gives the required size of the object in pixels,

$$N_{object} = \frac{2R\alpha\upsilon_{object}}{1.22\lambda}. \tag{11}$$

2 IMPLEMENTATION NOTES

2.1 WAVEFRONT AND PSF COMPUTATION

Equation 1 can be expressed in vector notation,

$$\mathbf{W} = \mathbf{c} \cdot \mathbf{Z}. \tag{12}$$

The first step in producing a wavefront is to create a matrix, $\mathbf{Z}$, containing the Zernike basis set, $Z_n^m(r,\theta)$. The Zernike matrix is constructed by creating an array of radial coordinates (between 0 and 1 along the

axes) and an array of angular coordinates (between $-\pi$ and π, with $\theta = 0$ along the positive horizontal axis). These are used to calculate the wavefront error associated with each Zernike mode using Equations 2, 3 and 4.

The matrix $\boldsymbol{Z}$ is 3-dimensional array of size (N_Z, a, a), where N_Z is the number of Zernike polynomials to construct the wavefront from and a is the number of pixels across the height (and width) of the wavefront. The first index of $\boldsymbol{Z}$ corresponds to the mode number. By default, Zernike modes are calculated starting from zeroth radial order (piston, Z_0^0) so that the index in the Zernike matrix is matched to the standard single index scheme (**Thibos et al.**, 2000). To avoid any ambiguity, the mapping between single index and double index values can be retrieved, as shown in the iPython notebook example. The vector, $\boldsymbol{c}$, containing the Zernike coefficients, c_n^m, is an array of length N_Z, which is matrix-multiplied by $\boldsymbol{Z}$ to give the wavefront error matrix, $\boldsymbol{W}$.

2.1.1 Number of pixels ($a \times a$) to use in the arrays It is important to have a sufficient number of pixels across wavefront to correctly sample it otherwise there will be aliasing artefacts in the PSF. The Maréchal criterion states that the maximum rms wavefront error for an optical system to be well-corrected is $\frac{\lambda}{14}$. We wish to specify the maximum gradient between adjacent pixels such that there is no significant wavefront error across them, suggesting that the wavefront is well sampled and smoothly varying. The gradient between neighbouring pixels can be quantified as an amplitude of tip (Z_1^1) or tilt (Z_1^{-1}) and for these two Zernike modes a rms wavefront error of $\frac{\lambda}{14}$ corresponds to a peak-to-valley wavefront error of $\frac{\lambda}{3.5}$. However, to be cautious we advise that the difference in wavefront error between neighbouring pixels be less than $\frac{\lambda}{7}$ and a function is provided to check this. As a rough guide, for the aberrations used in our experiment (0.6 μm rms of defocus, Z_2^0, coma, Z_3^1, or secondary astigmatism, Z_4^2) 512 x 512 pixels across the wavefront was sufficient to meet the criterion.

It is important to note that the number of pixels in the wavefront defines the field of view of the PSF. The aperture function (and therefore the wavefront) must be padded with zeros by at least a factor of two before being Fourier transformed to maintain Nyquist sampling in the PSF. The factor by which the wavefront is padded is the oversampling factor. The number of pixels in the PSF is proportional to the oversampling factor and the pixel scale is inversely proportional to it. Since the field of view of the PSF is the number of pixels multiplied by the pixel scale, it is entirely dependent on the original number of pixels in the wavefront. Increasing the oversampling factor simply reduces the pixel scale and keeps the field of view constant. Just as it is important to correctly sample the wavefront, it is also important to ensure that the field of view of the PSF is large enough, otherwise the PSF will be wrapped around in the array by the Fourier transform operation. We provide an additional function to check this, which calculates the total intensity in the edges of the array containing the PSF. If this value is larger than the bit depth of the intensity pattern with which the PSF will be convolved, the edges of the PSF will contribute a resolvable intensity change to the output. This means that the PSF has sufficient intensity in the edges of the array to consider that it may be wrapped and so the field of view, and therefore the number of pixels in the wavefront, is too small. In general we find that if the wavefront error gradient criterion, specified in the previous paragraph, is met then the field of view will be large enough, but it is prudent to check this.

As an additional note, unlike some FFT algorithms, the one we have used (numpy.fft.fft()) does not require that the array size is a power of 2, but the computation is more efficient is such an array size is used.

2.2 CONVOLUTION OF THE PSF WITH THE INPUT INTENSITY PATTERN

2.2.1 Constructing the input intensity pattern For a specified field of view, the input intensity pattern should be constructed with the appropriate number of pixels, as defined by Equation 11. If the pixel sampling is too high for the display, the output intensity pattern generated by the convolution should

be resized, taking care to apply any necessary (e.g. anti-aliasing) filters. It is also possible to match the pixel scale in the PSF to that in the input intensity pattern by choosing an appropriate oversampling factor, however it is more convenient to specify the oversampling factor as an integer (and preferably a multiple of 2 to maintain the power-of-2 array size) and to modify the number of pixels in the input intensity pattern. If the resulting number of pixels in the input intensity pattern is larger than the number of pixels in the PSF, the PSF can be padded with zeros to increase its field of view to match that of the input intensity pattern. If the resulting number of pixels in the input intensity pattern is smaller than the number of pixels in the PSF, the input intensity pattern should be constructed with a larger field of view and then the output from the convolution operation can be cropped to the required field of view. Alternatively, cropping the PSF so that its field of view matches that of the input intensity pattern is possible but care should be taken to check that the PSF is not clipped at the edges.

When creating the input intensity pattern, it is important to note that the Python module *numpy* uses C index ordering by default. That is, the first index of an array is the slowest changing dimension (typically the y-dimension in an image) and the second index is the fastest changing dimension (typically the x-dimension in an image). We also specify that the coordinate [0,0] is located in the top left-hand corner of the image. Care should be taken to make sure that the indexing scheme of the input intensity pattern matches that used in the array containing the PSF. Input intensity patters can be any data type. They will be converted to floating point for the convolution and then converted back to the original data type.

When performing the convolution, the arrays containing the PSF and the input intensity pattern are padded by a factor of 2 by default. This is to avoid edge effects due to wrapping in the Fourier transform. The PSF is padded with zeros, since the edges of the PSF should be zero, and the input intensity pattern is padded with mirrored copies of the original pattern, which avoids discontinuities at the boundaries.

2.2.2 Chromatic stimuli This basic version of the code considers only a monochromatic PSF and so three- or four-channel colour images should be converted to a single channel by whatever process is appropriate for the desired stimulus. The monochromatic PSF should not be used be used to make chromatic stimuli by applying a PSF to each colour channel independently. A polychromatic PSF must be constructed from an intensity-weighted linear sum of monochromatic PSFs calculated for each wavelength in the spectrum of the illumination, taking care to match the pixel scales across different wavelengths. This may only be convolved with a stimulus that is spectrally homogeneous. The red, green and blue phosphors of a CRT are individually spectrally homogeneous but when combined are spectrally inhomogeneous and so a separate polychromatic PSF must be considered for each channel in isolation (see **Ravikumar et al.**, 2008, for a discussion).

REFERENCES

Bedgood, P., Daaboul, M., Ashman, R., Smith, G., and Metha, A. (2008), Characterisitcs of the human isoplantic patch and implications for adaptive optics retinal imaging, *Journal of Biomedical Optics*, 13, 2, 1–7

Ravikumar, S., Thibos, L. N., and Bradley, A. (2008), Calculation of retinal image quality for polychromatic light, *Journal of the Optical Society of America A*, 25, 10, 2395–2407

Thibos, L. N., Applegate, R. A., Schwiegerling, J. T., and Webb, R. (2000), Standards for reporting the optical aberrations of eyes, in Vision Science and its Applications (Optical Society of America), SuC1
